# Risk maps for urban fire with geospatial model-based framework

**DOI:** 10.1038/s41598-026-38373-2

**Published:** 2026-02-07

**Authors:** Ke Wu, Sha Lu, Yishuo Jiang, Ming Chen, Jianing Luo, Liping Jiang, Tianhang Zhang, Yuxin Zhang, Xinyan Huang

**Affiliations:** 1 Key Laboratory of Offshore Geotechnics and Material of Zhejiang Province, Zhejiang University, Hangzhou, China; 2 Research Center for Urban Fire Safety Engineering, Zhejiang University, Hangzhou, China; 3 Department of Systems Engineering, Cornell University, Ithaca, USA; 4 School of Mechanical Engineering, Southwest Jiaotong University, Chengdu, China; 5 Fire Rescue Brigade of Xiaoshan District, Xiaoshan, China; 6 Dept. of Building Environment and Energy Engineering, Hong Kong Polytechnic University, Hong Kong, China

**Keywords:** Urban fire risk, Spatial autocorrelation, Regression, Urban planning, Fire risk management, Natural hazards, Civil engineering

## Abstract

Accurate identification of urban fire spatial patterns and governing factors is critical for optimizing firefighting resource allocation and developing sustainable cities resilient to evolving risks. Identifying spatial patterns of urban fires depends critically on the scale at which clustering is analyzed, yet a systematic approach to determine this optimal scale remains lacking. Moreover, the quantitative influence of fine-grained land-use structure on fire occurrence is not well understood. To overcome these gaps, this study proposes a novel three-stage framework for constructing hierarchical urban fire risk maps conditioned on built-environment macrostructure: (1) Investigation and selection of influencing factors, innovatively introducing high-resolution urban land-use attributes as variables, alongside traditional factors such as population density and socioeconomic indicators; (2) Determination of optimal grid size by integrating two key indicators: the Moran’s Index and Silhouette Score, ensuring precise spatial clustering. Subsequently, the spatial autocorrelation of urban fires is quantified; and (3) Negative binomial regression-driven risk quantification, deriving factor weights to calculate cell-level risk scores and generate hierarchical risk levels. Finally, a case study is conducted using fire data from Xiaoshan, China, a typical urban district with a population of about 2 million, covering 4,967 incidents from 2020 to 2023. The fire risk map indicates that urban fire distribution demonstrates striking conformity to the 80/20 rule, with a small share of cells concentrating most of the risk. This methodology provides stable, actionable risk mapping for strategic fire resource deployment and urban planning.

**Supplementary Information:**

The online version contains supplementary material available at 10.1038/s41598-026-38373-2.

## Introduction

Urban fire incidents have long been a subject of widespread concern globally due to their significant impact on human life, property safety, and the natural environment. According to the *International Fire Safety Standards* report, fires cause significant global harm, resulting in over 400 daily deaths and 19,000 injuries, which lead to profound physical, emotional, psychological, and financial suffering. The economic impact is equally severe, with direct damages from fire incidents estimated to account for nearly 1% of global GDP, a cost that encompasses infrastructure destruction, business losses, and service interruptions^1^. This global pattern is reflected at the national level: the China Fire and Rescue Bureau reported 825 thousand fire incidents in 2022, resulting in 2,053 deaths and 2,022 injuries, with direct property losses of approximately $1.0 billion. The US National Fire Protection Association (NFPA) reported around 1.5 million fire incidents in 2022 which caused 3,790 deaths and 13,250 injuries, with a financial toll of around $18 billion^2^.

Given this severe and widespread impact, proactive and efficient risk mitigation is paramount, which necessitates a deeper understanding of fire patterns themselves. Accurate identification of the spatial distribution characteristics and governing factors of urban fire incidents is crucial for optimizing firefighting resource allocation^3^. Tobler’s First Law of Geography states, “Everything is related to everything else, but near things are more related to each other.”^4^ Fires, as events that occur within specific spatiotemporal contexts, exhibit strong spatial autocorrelation: a characteristic where variables at specific locations tend to show similar (positive autocorrelation) or dissimilar (negative autocorrelation) values relative to one another, beyond what would be expected in a random spatial pattern. The clustering phenomenon has been validated both in urban fire and forest fire. For instance, in the Yeguare Region of Honduras, Caceres^5^ adopted remote sensing and the Getis-Ord Gi* algorithm, revealing a significant concentration of fire hotspots primarily in forest and rangeland areas. Similarly, Baykal et al.^6^ found that Moran’s index performed best overall in detecting forest fire hotspots in Türkiye through a systematic comparison of three commonly used spatial clustering methods. Parallel findings emerge from research on urban fire regimes. Chen et al.^7^ collected fire incident data from Tainan City in southern Taiwan and found that areas characterized by high population density and economic development face elevated fire risks. Further, Xiong et al.^8^ utilized spatial correlation analysis across provinces in China from 1999 to 2019. The results indicate that although China’s overall fire situation improved over this twenty-year period, the spatial clustering of fires became increasingly prominent. However, despite the widespread recognition of spatial autocorrelation in fire distributions, a critical unresolved question remains: at what spatial scale should fire clustering be analyzed to most effectively reveal meaningful patterns? With the exception of Xiong et al.^8^, who used provincial boundaries as their de facto analytical grid, the other cited studies relied on predetermined grid sizes without quantitatively justifying their selection or determining the optimal scale. This common practice fails to systematically address whether the chosen scale best captures the inherent spatial structure of fire events, potentially introducing statistical noise or excessive aggregation that undermines the precision of risk maps.

Furthermore, although demographic and socioeconomic factors are widely recognized as key determinants of fire occurrence, human activities of similar intensity can lead to significantly different fire risks across various urban functional zones. This divergence stems from the distinct nature of activities in different zones. For instance, residential areas are characterized by cooking and electrical appliance usage, while industrial zones involve more open flame operations during production processes, resulting in distinctly different fire risks. However, existing research has yet to fully elucidate the explanatory power of urban land-use structure in accounting for fire variance.

To address these dual deficiencies, this study hypothesizes that land-use composition significantly explains fire risk variation and validates this through a three-stage framework: (1) Investigation and selection of influencing factors, innovatively integrating high-resolution urban land-use attributes with traditional variables (population density, socioeconomic indicators); (2) Determination of optimal grid size through joint optimization of Moran’s Index (spatial autocorrelation quantification) and Silhouette Score (cluster cohesion validation); and (3) Negative binomial regression-driven risk quantification, deriving factor weights to calculate cell-level risk scores and generate hierarchical risk levels conditioned on built-environment macrostructure. Applied to 4,967 fire incidents (2020–2023) in Xiaoshan, China, a region whose demographic and economic indicators—including its 81.3% urbanization rate and diverse industrial base—are representative of many other rapidly developing, mid-sized to large Chinese cities. The framework subsequently generated stable and actionable risk maps to guide strategic fire resource deployment and urban planning.

## Literature review

**Fig. 1:**
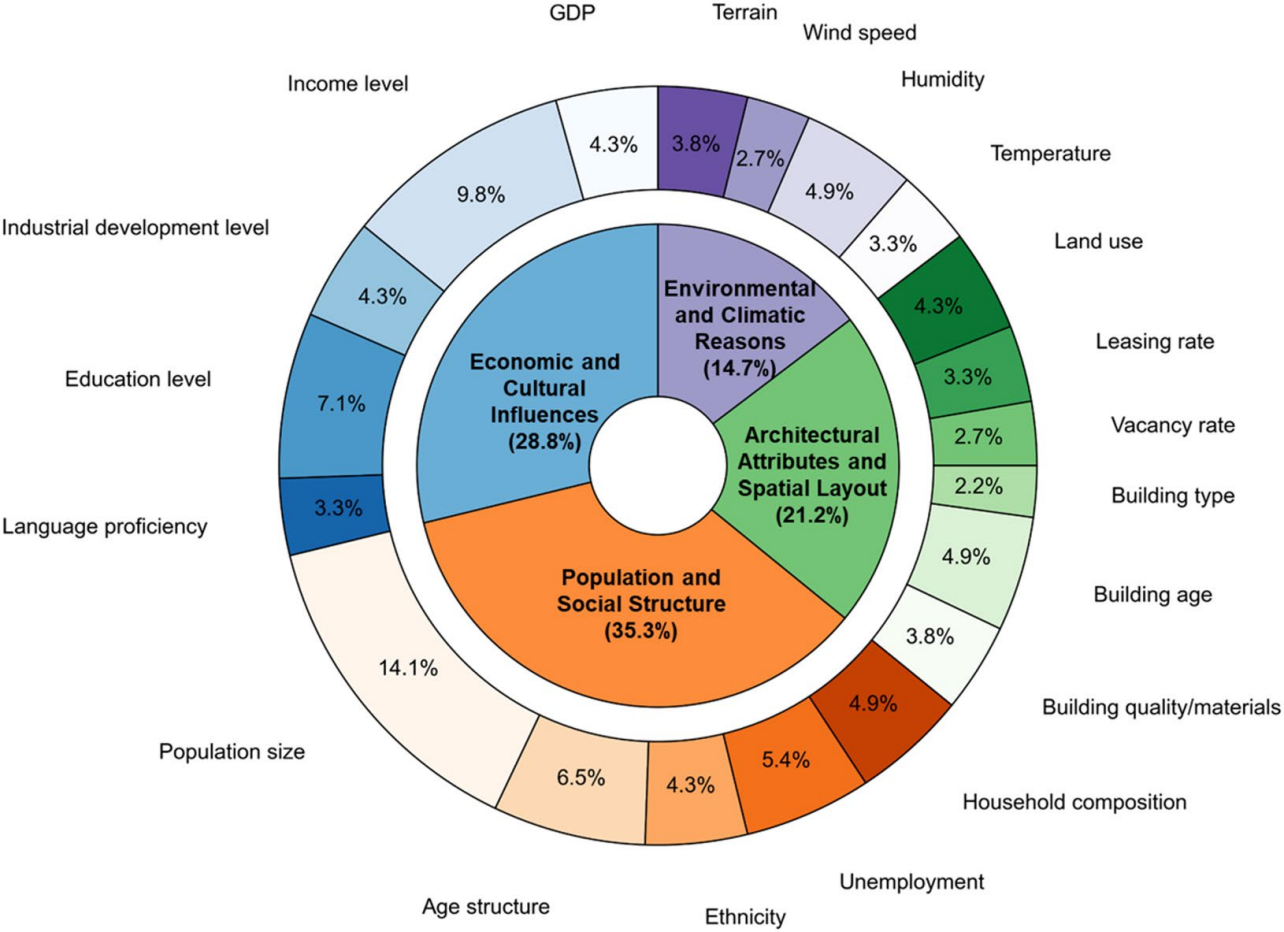
A systematic quantification of factors influencing urban fire distribution.

This literature review will first discuss existing studies on the influencing factors of urban fires, followed by an examination of the methodologies employed in these studies. Urban fires, as socially driven phenomena, exhibit spatial clustering influenced by numerous social factors, which are distinctly different from the influences on forest fires. Figure 1 quantifies the relative citation frequency of various influencing factors based on a systematic review of existing literature. As shown, the factors affecting the spatial distribution of urban fires can primarily be categorized into four types: (1) Economic and Cultural Influences, (2) Population and Social Structure, (3) Architectural Attributes and Spatial Layout, and (4) Environmental and Climatic Reasons. Among these, Economic and Cultural Influences and Population and Social Structure are the most frequently cited categories (collectively accounting for 64.1%), Architectural Attributes and Spatial Layout is cited in 21.2% of cases, while Environmental and Climatic Reasons constitutes only 14.7%. This indicates that urban fires are primarily driven by anthropogenic and socio-economic environments. This figure provides an intuitive, literature-based foundation for the subsequent selection and integration of key variables in this study.

*Economic and cultural influences* include factors related to economic conditions and cultural practices, e.g., GDP, per capita income level, industrial development level, language proficiency, education level. As synthesized by Jennings^9^, scholars in the 20th century examined the correlation between fire risk and socioeconomic variables aggregated by administrative divisions, consistently finding a significant negative association between income, education, and the incidence of fires. Duncanson et al.^10^ found that the rate of fatal unintentional domestic fire incidents in the most socioeconomically deprived meshblocks was 5.6 times higher than in the least deprived areas in New Zealand. Wang et al.^11^ analyzed fire data from 283 prefecture-level cities in China from 2000 to 2009 and found that a 1% increase in GDP per capita would lead to a 0.15% decrease in the average fire incidence in China. Based on an analysis of 324 neighborhoods in Philadelphia, Shai^12^ indicated that low income is often associated with poor-quality housing and aging infrastructure, which increases the risk of fire and casualties. Corcoran et al.^13^ categorized urban fire incidents into four types: structural fires, vehicle fires, outdoor fires, and false alarms. They found that areas with lower levels of education are more susceptible to structural and outdoor fires while regions with higher vehicle ownership rates are more prone to vehicle fires and false alarms. As for the factor of education level, existing studies generally believe that a higher average education level corresponds to a lower urban fire incidence^3,14–16^.

*Population and social structure* cover population density, age structure, unemployment rate, ethnicity, household composition, etc. In general, regions with higher population densities tend to exhibit elevated rates of fire incidence^3,14,17–19^. Shai^12^ and Hastie & Searle^20^ argued that being unemployed or on extended sick leave raises the likelihood of fires happening. By analyzing the statistical local areas (SLA) in Australia, Chhetri et al.^21^ found a positive relationship between a higher proportion of indigenous population and increased building fire incidence. Duncanson et al.^10^ emphasize that children of single mothers face a higher fire fatality rate, with the increased risk associated with a lack of adequate supervision, poor housing conditions, and higher smoking rates. Anderson-Bell et al.^14^ incorporated crime rates into their examination of urban fire influences, revealing that areas with higher levels of social instability also exhibit increased fire risks.

*Architectural attributes and spatial layout* encompass factors such as building age, type, leasing rate, vacancy rate, and urban land use. This category focuses on the crucial role of physical infrastructure and spatial urban design in urban fire risk. Crowded building layouts^13,17,22^ and low-quality building materials^10,19^ are widely recognized as risk factors for urban fires. Apart from that, Duncanson et al.^10^ found that residential dwellings represent the highest fatality rates in fires, with older houses posing significantly elevated electrical fire risks due to poor maintenance. Shai^12^ and Buffington et al.^23^ discovered that vacant houses present potential fire hazards in urban areas. The primary causes of fires in vacant houses are deliberate arson and open flames, often attributed to the presence of drug users and homeless individuals within these properties. Additionally, Lim et al.^24^ explored the relationship between the area of different land types and the number of fires in 273 administrative areas in South Korea. The regression analysis indicates that residential areas have a positive impact on the number of fires while commercial and green areas have a negative impact.

*Environmental and climatic reasons* emphasize the importance of natural conditions such as humidity, wind, temperature and precipitation to the occurrence of urban fires. Wang et al.^11^ found that a 1% decrease in annual average humidity would result in a 0.99% increase in the average fire incidence rate in China. Liu et al.^25^ collected and processed a dataset comprising 20,622 fire incidents along with historical weather data from Changsha, China. Through data mining, they discovered that the average daily fire frequency tends to be lowest within the temperature range of (20 °C, 25 °C], which is associated with lower electricity utilization rates. Gibson et al.^26^ found that medium wind speeds (1.6–7.9 m/s) were associated with the largest and most destructive residential fires. However, higher wind speeds (> 8 m/s) reduced the frequency of large fires, likely due to reduced flame height and increased convective cooling, which delayed ignition and flashover in adjacent dwellings.

These influencing factors and spatial granularity in existing studies are summarized in Table 1. As shown in Table 1, existing studies are largely confined to the administrative division level, which fails to provide the hundred-meter‑scale resolution required for optimizing the allocation of urban firefighting resources. Moreover, these studies predominantly rely on retrospective statistical indicators—such as socio‑economic data collected after urban construction—which cannot offer forward‑looking guidance for land‑use design during the urban planning stage. Although Lim et al.^24^ have preliminarily examined the relationship between land‑use attributes and fire frequency, their analyses remain at the macro‑scale of administrative units and thus cannot reveal the underlying mechanisms at finer spatial resolutions. To address these gaps, this study introduces, for the first time, “fine‑grained urban land‑use structure” as a key independent variable alongside conventional socio‑economic factors. Using a negative binomial regression model, we quantify its specific impact on urban fire risk. Based on the weight of each influencing factor, a spatial fire‑risk map is generated, providing a novel quantitative basis for urban fire‑risk stratification and the spatial arrangement of prevention resources.

**Table 1:** Influencing factors and study methods of urban fire.

Reference	Country/Region	Granularity	Indicators
^16^	China	Province level	Per capita GDP, Education
^8^	China	Province level	/
^3^	China	Prefecture level	Population size, Population density, Income level, Per capita GRP, Consumption capacity, Industrial Development level, Education level
^11^	China	Prefecture level	Annual average temperature, Annual average relative humidity
^27^	China	City level	Daily minimum air temperature, Daily maximum air temperature
^24^	South Korea	City level	The land use area
^20^	the UK	Community level	The proportion of residents who have not worked for more than five years or have never worked, The proportion of single person households where the resident is aged under 65
^10^	New Zealand	Community level	Communication, Income, Employment, Transport, Support, Owned home, Living space
^12^	the US	Community level	Older housing (prior to 1940), Low income, The prevalence of vacant houses, The ability to speak English
^15^	Australia	Community level	Car ownership, Educational attainment, Ethnicity, Household structure, Family structure, Age profile, Housing tenure
^17^	Australia	Community level	The Index of Relative Socio-economic Advantage and Disadvantage (IRSAD), Residential density, Housing tenure and mobility
^23^	United States	Community level	Older housing units, Low income, Low education level, Vacancy rate
^26^	South Africa	Community level	Wind speed, Wind direction, First nearest neighbour distance, Edge density, Fire extent area
^18^	Australia	Hundred-meter scale	Population, Number of residential dwellings
^28^	China	Hundred-meter scale	Population, Precipitation, Relative humidity, Sunshine duration, Temperature, GDP, The average housing area

## Methods

### Study area and data source

**Fig. 2:**
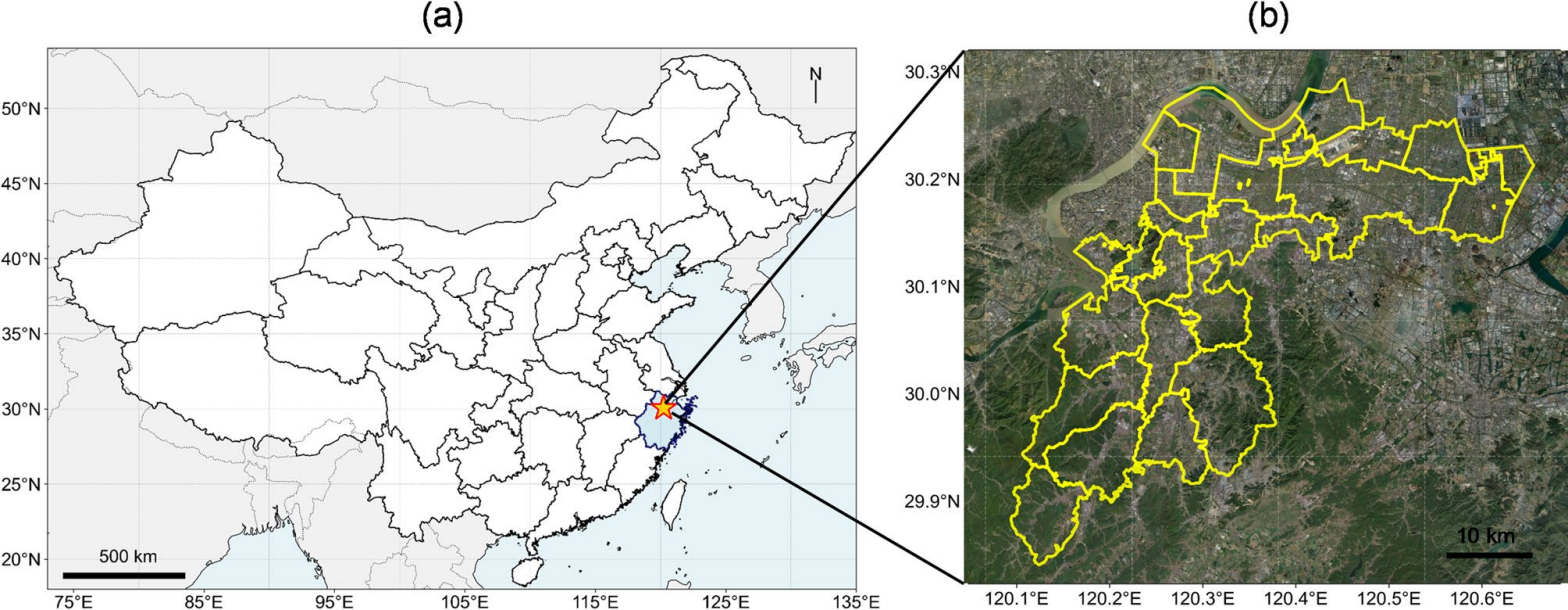
**(a**) The location of Xiaoshan District in China, and (**b**) administrative boundaries of Xiaoshan District’s geographic coordinates.

The target area is Xiaoshan District, which is located in southeast of China and serves as a significant economic and transportation hub, having hosted major international events such as the 19th Asian Games and the G20 Summit (Fig. 2). Covering an area of 931 km² with a population exceeding 2 million, Xiaoshan is home to one of China’s largest cargo airports and stands as a major industrial base in Zhejiang Province. The district hosts multiple industrial parks with diverse industries, including machinery, electronics, chemical engineering, and food processing. This economic prosperity and dense population contribute to a heightened risk of fire incidents, which is a representative sample for the modern city. The dataset used in this study was sourced from the Xiaoshan District Fire and Rescue Brigade under a data sharing agreement for research purposes. It consists of 4,967 anonymized fire incident records, covering the period from January 2020 to August 2023. The dataset includes geographic coordinates (latitude and longitude, geocoded from addresses), the date and time of each incident, and the primary cause classification. All personal and sensitive identifiers were removed by the data provider prior to acquisition.

### Grid size sensitivity analysis

The grid size (spatial granularity) determines the level of detail(s) available in the analysis. Typically, a smaller grid size allows for finer spatial resolution, capturing local variations more precisely. Nevertheless, statistical noise and overfitting will be introduced if the grid size is too small, such that random variations are mistaken for significant patterns. When setting the grid size for spatial analysis, several critical aspects must be considered to ensure that the analysis is both meaningful and accurate. First and foremost, it is crucial to determine the scale level required for urban planning and management, which directly determines variables that need to be captured.

Additionally, a suitable grid setup is one that minimizes the occurrence of empty cells and prevents the aggregation of excessive data within single cells. Another important consideration is that the grid size should be consistent with the scale of other relevant datasets to facilitate coherent spatial analysis and interpretation. In this work, in addition to the GMI and LMI distribution, *Silhouette Score* is also adopted to estimate the quality of clustering to support the grid size sensitivity analysis (see Supplementary Material). The approach to selecting and evaluating grid size forms the bedrock of spatial analysis, guaranteeing that the chosen grid size is ideally suited for elucidating and interpreting spatial patterns pertinent to the objectives.

### Spatial analysis method based on GMI and LMI

Drawing on the foundational concept of spatial autocorrelation articulated by Tobler’s First Law of Geography, this section examines the quantitative methods used to analyze spatial patterns in urban fire incidents. Spatial autocorrelation—measuring how a location’s attributes correlate with those of nearby locations—serves as a key metric in identifying spatial clustering and distribution trends. This study applies the Global Moran’s Index (GMI) and Local Moran’s Index (LMI) to quantitatively assess these spatial dynamics, each providing distinct insights into the clustering and spatial behavior of urban fires.

The GMI is a statistical measure that quantifies spatial autocorrelation by considering both the geographic locations and attribute values of the features under study. GMI values range from -1 to 1, namely, a GMI greater than 0 indicates positive spatial correlation, suggesting that similar values are clustered in space; a GMI less than 0 indicates negative spatial correlation, reflecting a dispersion of similar values; and a GMI near 0 suggests a random spatial distribution. The GMI is mathematically defined as follows:

1 $$\:GMI=\frac{n{\sum\:}_{i}{\sum\:}_{j}{w}_{ij}\left({X}_{i}-\stackrel{-}{X}\right)\left({X}_{j}-\stackrel{-}{X}\right)}{\left({\sum\:}_{i\ne\:j}{w}_{ij}\right){\sum\:}_{i}\left({X}_{i}-\stackrel{-}{X}\right)}$$

where *X*_*i*_ and *X*_*j*_ represent the fire counts in adjacent paired spatial cells; *w*_*ij*_ is the spatial weight matrix (*n* × *n*), and in this study the weights are defined by a Gaussian distance-decay kernel function, *w*_*ij*_ = exp(-*d*_*ij*_^2^), where *d*_*ij*_ is the normalized Euclidean distance between the centroids of grid cells *i* and *j*; $\:\stackrel{-}{X}$ is the mean value of the attribute across all observations.

The LMI, in contrast, is designed to identify local spatial autocorrelation, revealing clusters of similar values, known as “hot spots” and “cold spots”. These localized patterns provide insight into specific areas of interest within the broader study area, allowing for a more nuanced understanding of spatial dynamics. The LMI is particularly useful for pinpointing areas where urban fires cluster, facilitating targeted analysis and intervention. The LMI could be calculated as:

2 $$\:{LMI}_{i}=\frac{{X}_{i}-\stackrel{-}{X}}{{S}^{2}}\:\:{\sum\:}_{j\ne\:i}{w}_{ij}\left({X}_{j}-\stackrel{-}{X}\right)$$

where *LMI*_*i*_ represents the local Moran’s Index for the *i*-th geographical unit, *S*^2^ is the variance of local observed values, and the meanings of the remaining parameters are consistent with those of GMI. *LMI*_*i*_ > 0 indicates a region with high (low) values is enveloped by neighboring areas with similarly high (low) values, thus forming a hot spot (or cold spot), corresponding to a High-High (Low-Low) association. Conversely, points with *LMI*_*i*_ < 0 denote that a region characterized by high (low) values is encircled by areas with contrasting low (high) values, illustrating a High-Low (Low-High) association.

It’s worth noting that both indices require consideration of spatial weights, which capture the spatial relationships between units within the study area. Calculating these indices involves comparing the observed spatial distribution against a random distribution to evaluate the significance of spatial autocorrelation. A hypothesis test is commonly used to assess spatial autocorrelation, with the null hypothesis positing that the observed data are randomly distributed across space. If the p-value is below a specified threshold (e.g., 0.05), the null hypothesis is rejected, confirming statistically significant spatial autocorrelation. In this study, the application of GMI and LMI facilitates a thorough examination of the spatial characteristics of urban fires. These methods capture both global trends and local anomalies, providing a robust framework for understanding spatial dynamics. The resulting insights are valuable for informing urban planning and enhancing emergency response strategies.

### Selection and analysis of influencing factors for urban fire

To overcome the limitations in existing studies, this work selects indicators that are essential not only for interpreting urban construction outcomes but also for proactive fire mitigation during the planning phase. As highlighted in the introduction, most prior studies rely on post-construction indicators, which offer limited guidance for preventative planning. In response, the proposed framework prioritizes planning-oriented, fine-grained land-use data, and combines it with traditional socioeconomic data—population density and GDP—that jointly shape urban fire risk and can inform preemptive planning strategies. Given that the study area is predominantly flat plain and covers a limited extent, meteorological variables exhibit negligible spatial heterogeneity at the intra-district 500-m scale of this cross-sectional analysis. Therefore, climate variables were not included here.

This proposed framework employs four distinct datasets to evaluate the effectiveness of the methods and algorithms presented: (a) fire incident records, (b) land-use classifications, (c) population density and (d) GDP. The fire incident dataset, sourced from the local Fire Department, captures comprehensive details for each occurrence, including the time, location, cause of the fire, and the consequent economic loss. The land use dataset, obtained from the local Natural Resources Bureau, categorizes the area into six land use types: residential, commercial, public administration and service, transportation, industrial, and undeveloped land. Meanwhile, the population density information has been acquired from the WorldPop Hub—an accessible online data platform at https://hub.worldpop.org/geodata. GDP is derived from a publicly available gridded dataset^29^. The specific explanations of different factors are listed in Table 2.

**Table 2:** The interpretation of factors influencing urban fire Incidents.

Classification	Interpretation
Population density	The number of people per square kilometer
GDP	GDP within each grid (in millions)
Residential land	Land for residential housing and accompanying facilities used for people’s living
Commercial land	Land for commercial offices or business and trade services
Transportation land	Land for urban roads, highways, and vameirious transportation stations
Industrial land	Land for production, warehousing, mining, and construction installations
Public administration and service land	Land used for public service institutions and organizations, including schools, hospitals, libraries, museums, etc.
Undeveloped land	Include forests, fields, vacant lots, or other areas that have not been subjected to urbanization or construction activities

### Regression method

Regression analysis is a powerful statistical tool used to analyze and predict relationships between variables. It identifies and quantifies associations between independent (predictor) and dependent (outcome) variables, enables the forecasting of dependent variable values based on given independent values, and assesses the strength of these relationships. Commonly used generalized linear models include multiple linear regression, Poisson regression, and negative binomial regression. Since fire incident data are *non-negative count* data, we assume a probability distribution for the dependent variable suited to non-negative integers, such as the Poisson or negative binomial distribution. Therefore, Poisson and negative binomial regression models are especially appropriate for the present scenario.

When an event exhibits a “memoryless” property—meaning the probability of occurrence in any region is independent of the time since the last occurrence in that area—the Poisson distribution may be appropriate. This approach assumes that individual events are mutually independent. However, a key requirement of the Poisson distribution is equidispersion, where the mean and variance of the data distribution should be equal. In our analysis, however, fire incidents show substantial clustering, with a mean of 1.2 and a variance of 7.8 across grid cells, indicating a variance much greater than the mean value. This overdispersion contradicts the Poisson distribution’s assumptions, making it unsuitable for the present scenario.

The negative binomial distribution extends the Poisson distribution to better manage instances of overdispersion in data by incorporating an additional parameter. This enhancement provides the negative binomial distribution with greater flexibility compared to the Poisson distribution, making it particularly adept at accommodating data characterized by significant variance. Consequently, the Negative Binomial Regression (NBR) model emerges as a more effective tool than its Poisson counterpart for analyzing overdispersed data, offering enhanced adaptability to various patterns of data volatility. Given these advantages, this study selects the Negative Binomial Regression Model to investigate the effects of population density and land use types on the incidence of urban fires. NBR refers to:

3 $$\:{ln}\left(y\right)={\beta\:}_{0}+{\beta\:}_{1}{x}_{1}+{\beta\:}_{2}{x}_{2}+{\beta\:}_{3}{x}_{3}+\dots\:+{\beta\:}_{p}{x}_{p}$$

where *y* is the predicted variable, *x*_*i* (*i* = 1, 2, …, *p*)_ are the independent variables, and *β*_*i* (*i* = 0, 1, …, *p*)_ represent the regression coefficients to be estimated.

For datasets exhibiting a high proportion of zero counts, alternative specifications such as zero-inflated count models are sometimes employed to distinguish between structural zeros (inherently zero-risk areas) and sampling zeros (areas with potential risk but zero observed incidents). the Zero-Inflated Negative Binomial (ZINB) model assumes that the data-generating process comprises two distinct components:

1. A binary process determining whether an observation is a structural zero;
2. A count-generating process (modeled via a Negative Binomial distribution) governing the number of incidents when the observation is not structurally zero.

Formally, the ZINB probability mass function can be expressed as:

4 $$p\left( {Y=y} \right)=\left\{ \begin{gathered} \uppi +\left( {1 - \uppi } \right)NB\left( {0|\upmu ,k} \right),y=0 \hfill \\ \left( {1 - \uppi } \right)NB\left( {y|\upmu ,k} \right),{\text{ }}y>0 \hfill \\ \end{gathered} \right.$$

where 𝜋 denotes the probability of a structural zero, 𝜇 is the mean of the NB component, and 𝑘 is the dispersion parameter. The structural-zero component is typically modeled using a logit function and the count component retains the standard negative binomial regression form.

## Results

To address the three shortcomings identified in previous research: (1) lack of prospective parameters, (2) insufficient research granularity, and (3) inadequate methodological rigor, this paper proposes the framework to analyze spatial distribution characteristics of urban fires and their influencing factors. The basic process of the framework first involves the spatial segmentation of the study area, using a reasonable grid size to precisely analyze the spatial distribution of urban fires (Sect. 4.1). Then, the global Moran’s Index is used to assess the spatial autocorrelation of fire incidents within the entire study area, while the local Moran’s Index is employed to identify specific spatial clusters (Sect. 4.2). The next step of the framework considers the selection and analysis of influencing factors, including quantifiable variables from the urban planning stage and after the completion of urban construction that are closely related to the occurrence of fires (Sect. 4.3). Finally, the negative binomial regression model is adopted to evaluate how these factors affect the frequency of the fire incident (Sect. 4.4). To validate the proposed analytical framework, a case study is conducted in the Xiaoshan District of Hangzhou.

### Spatial aggregation pattern of fires

**Fig. 3:**
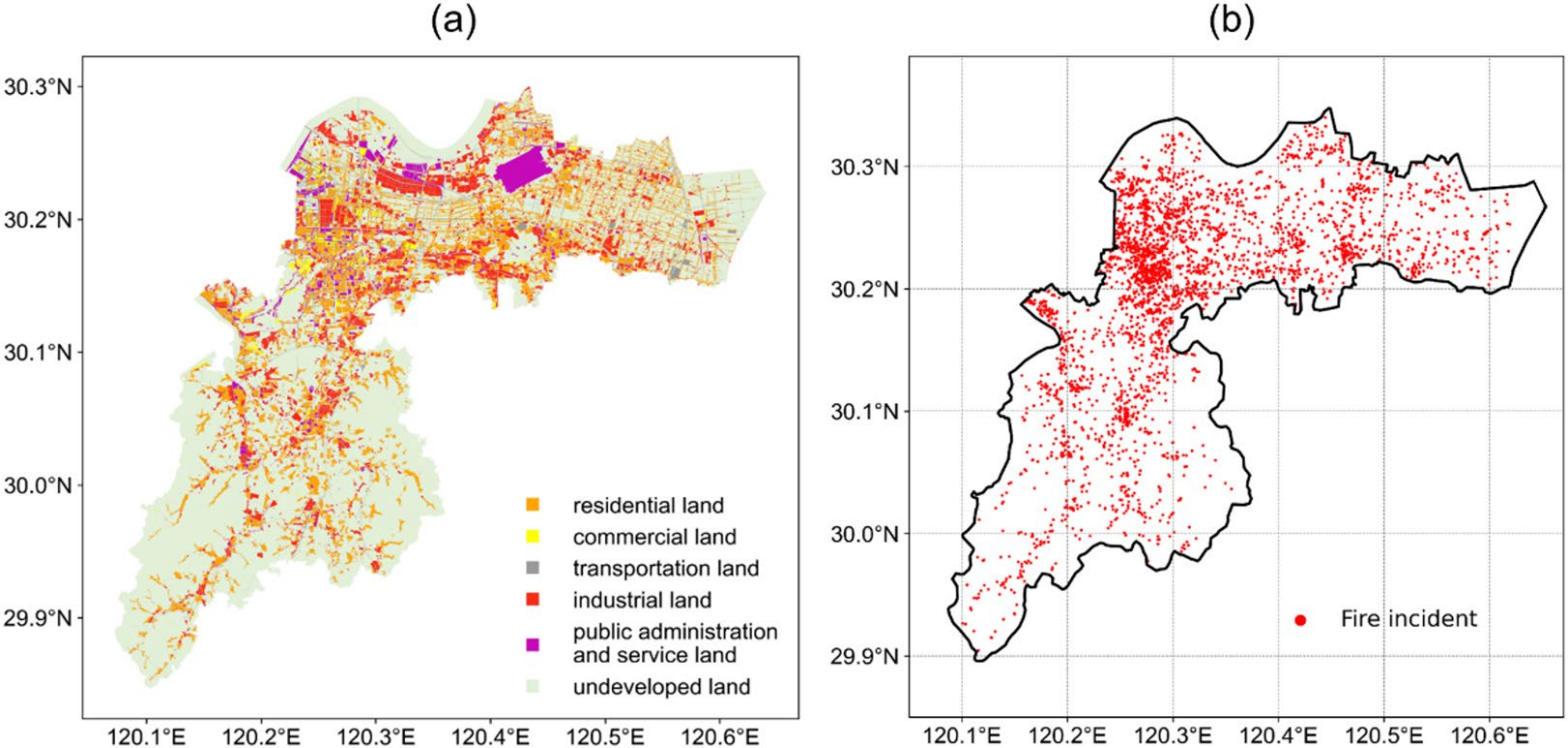
(**a**) The land use, and (**b**) The spatial distribution of fire incidents of the target area.

The spatial distribution of land use and fire incidents in the target area is plotted in Fig. 3. The various land use categories, including residential, commercial, transportation, industrial, public administration and service land, as well as undeveloped land are illustrated in Fig. 3a. Each category is color-coded, with the northwest part of the map representing the central urban area, characterized by diverse and highly developed land use. Figure 3b depicts the spatial distribution of fire incidents, marked by red dots. From a rough view, the fire events are predominantly concentrated in the central urban area, aligning with regions of high land use complexity and development. Additionally, smaller clusters of fire incidents are scattered throughout the district, indicating localized areas of fire activity.

**Fig. 4:**
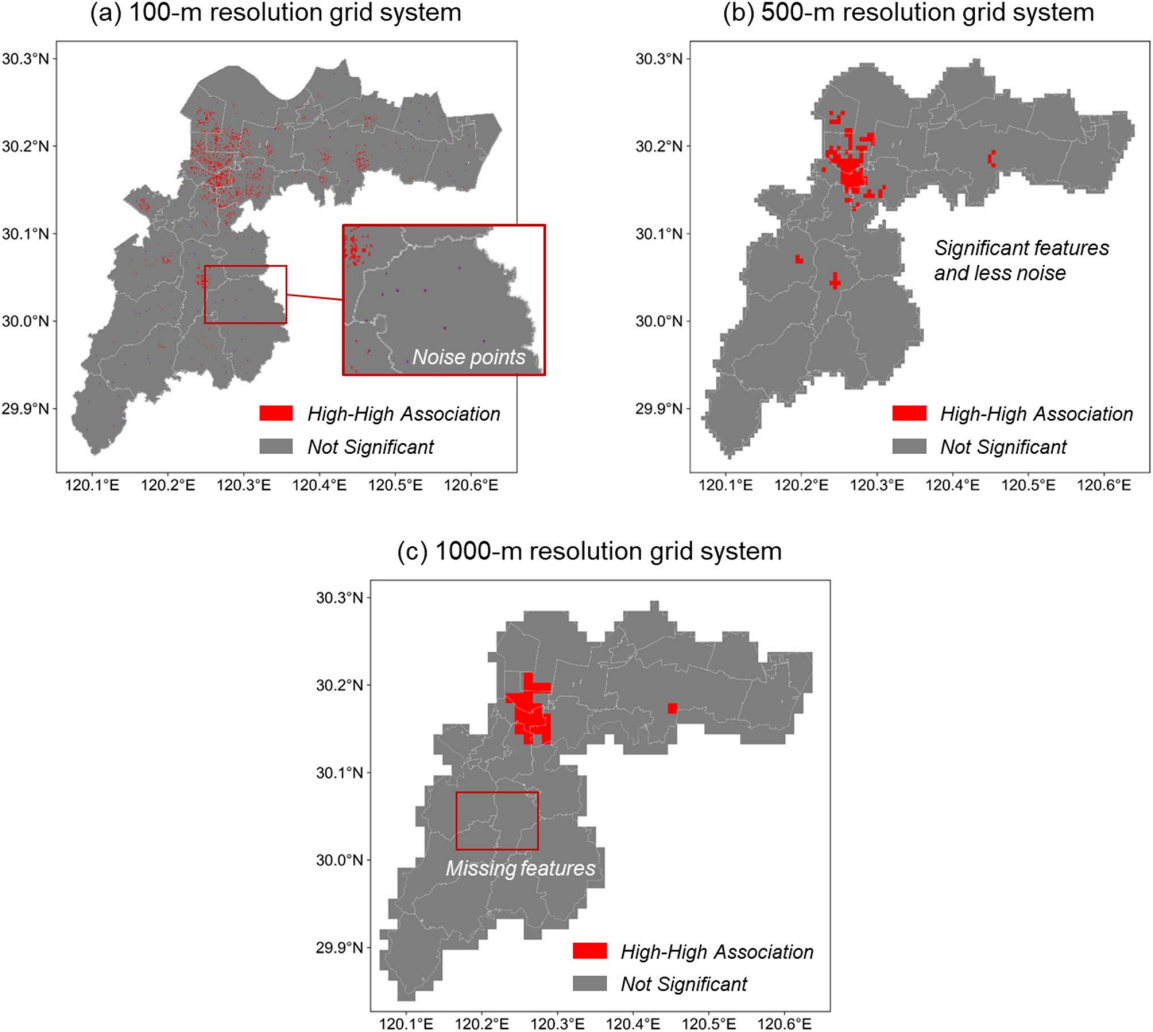
LMI distribution with various grid sizes (**a**) 100 m, (**b**) 500 m, and (**c**) 1000 m.

The selection of the spatial granularity plays a significant role when analyzing the fire incident spatial characteristics. For instance, as shown in Fig. 4a, the 100-m resolution grid system captures nearly all spatial details of fire distributions but also introduces noise and random variations, complicating the interpretation of results. In contrast, a 1000-meter resolution grid system effectively suppresses noise but obscures key local hotspots, thereby hindering precise identification and analysis. A grid size of 500 m appears to offer a balanced compromise whereas further quantitative evidence is still required to confirm its effectiveness.

**Fig. 5:**
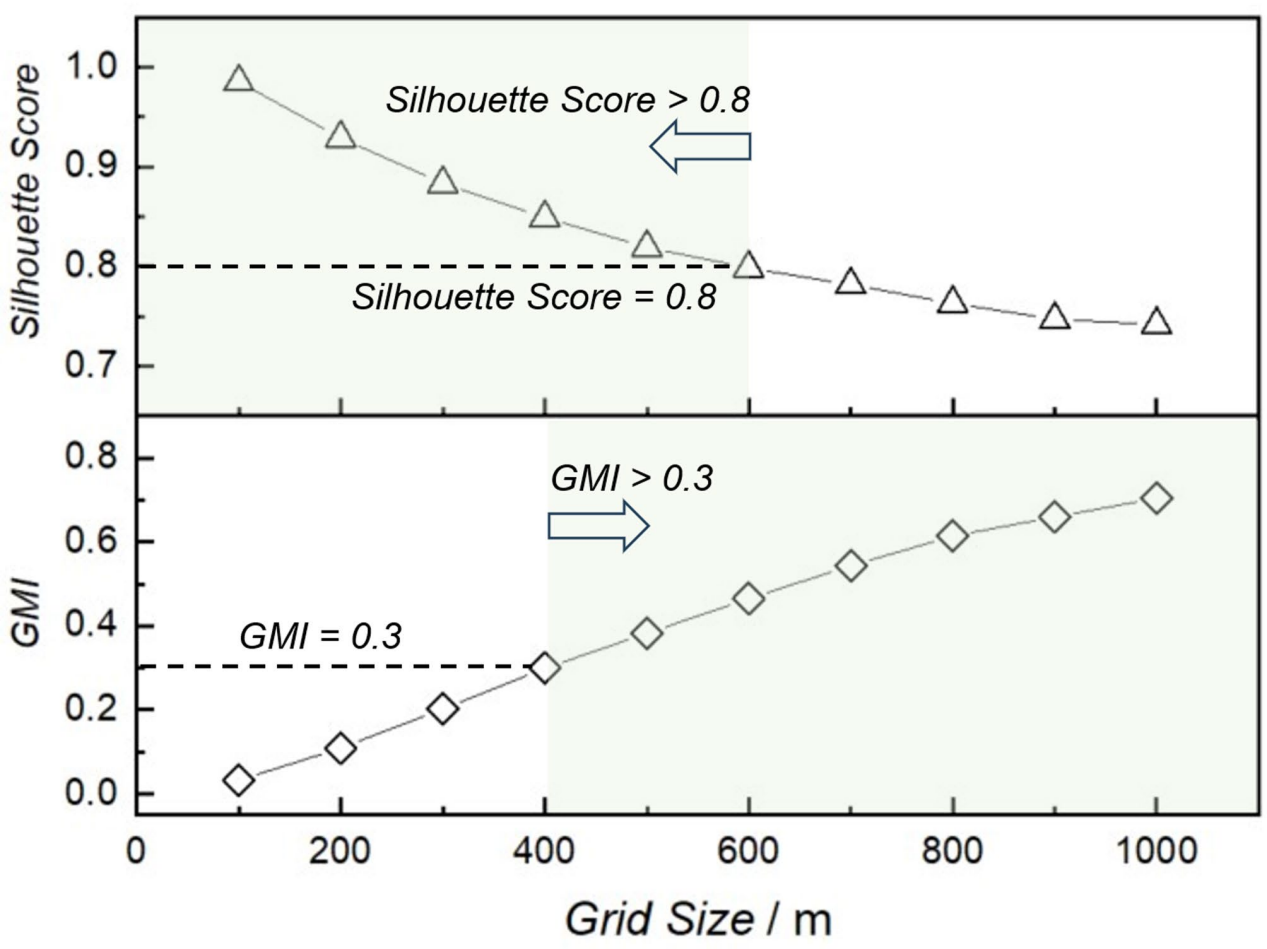
The variation of GMI and Silhouette score with the grid size.

The variations of the selected indicators (i.e., the GMI, and Silhouette Score) across different grid sizes are calculated and plotted in Fig. 5 to facilitate a detailed analysis of grid size sensitivity. As observed, the GMI value increases with the grid size, indicating that larger grids effectively capture the overarching spatial distribution of fire incidents while smaller grids are more susceptible to noise. Following common practice, we use *GMI* = 0.3 as a lower bound for non-random clustering^30^. Conversely, the Silhouette score decreases dramatically with the grid size. This trend arises because excessively large grids amalgamate finer spatial features into broader units, leading to poor clustering performance. Previous studies regard *Silhouette score* ≥ 0.7 as indicative of good clustering quality^31^. To be conservative, we tighten this requirement and adopt *Silhouette score* ≥ 0.8 as our working cutoff. Considering both spatial characterization and clustering effects, the optimal grid size range is determined to be between 400 and 600 m. Consequently, the 500-meter grid size employed in Fig. 4 represents a reasonable choice, balancing strong spatial clustering with high clustering quality.

Once the spatial granularity is established, the spatial autocorrelation of fire incidents in the target region can be analyzed, as shown in Fig. 4b. Four categories are first defined to describe the autocorrelation type of the fire incident, as summarized in Table 3. High-High and Low-Low areas are “hot spots” and “cold spots,” respectively, while Low-High and High-Low areas represent “outlier” points. Notably, all grid units surpassing the 95% confidence level fall into the “High-High” category, while the remaining grids exhibit a “not-significant” pattern. This suggests that fire distribution in Xiaoshan District is characterized by numerous “hotspots,” with an absence of “cold spots” or “outlier” points. The identified hotspots are primarily concentrated in the central urban area of Xiaoshan, the industrial park in the east, and two commercial centers in the southwest. These regions share common characteristics, such as high population density or specific land uses like industrial or commercial zones.

**Table 3:** Categories of Spatial autocorrelation for fire Incidents.

Label	Description	Explanation
High-High	Both a unit and its neighbors have high rates of fire incidents	Hot Spots
Low-Low	Both a unit and its neighbors have low rates of fire incidents	Cold Spots
High-Low	High incident rate units are surrounded by low incident rate units	Outliers
Low-High	Low incident rate units are surrounded by high incident rate units	Outliers

**Fig. 6:**
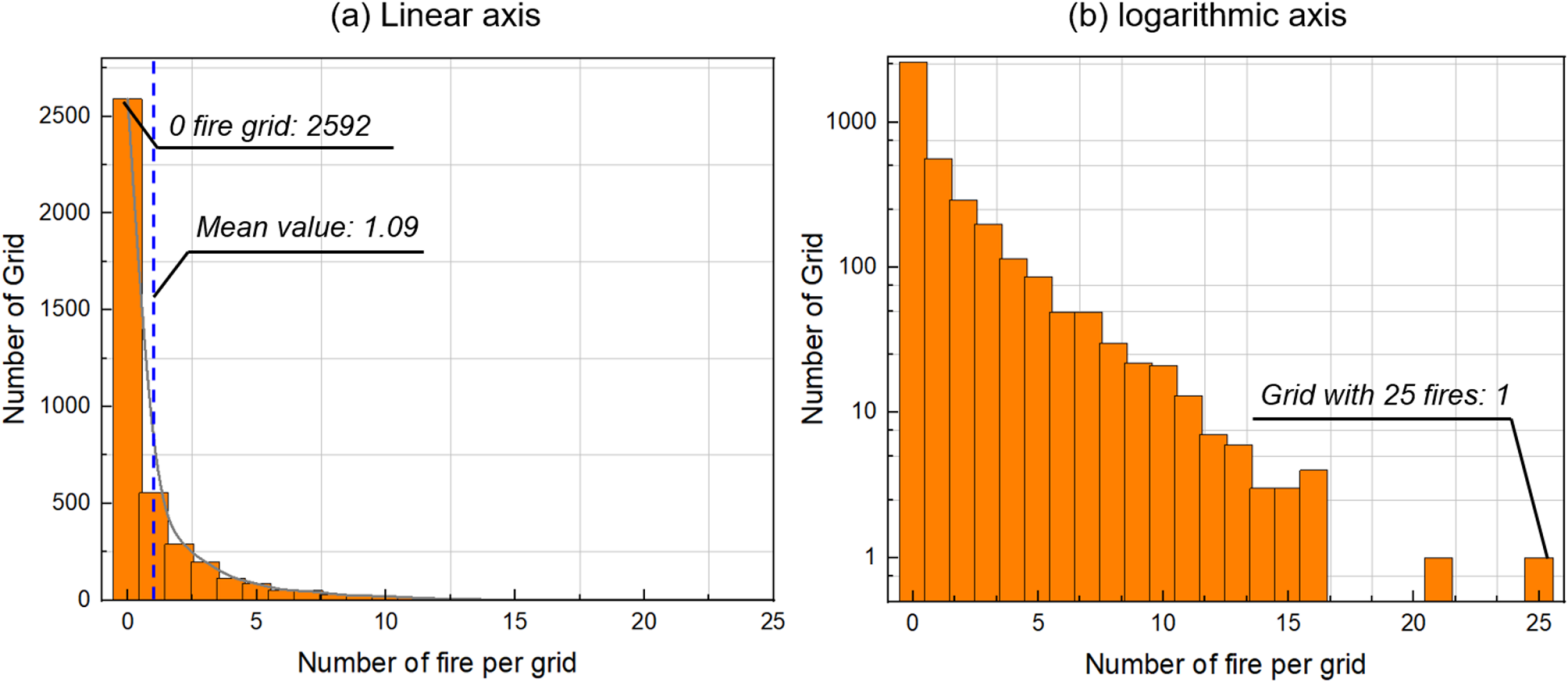
Distribution of Fire Frequencies in Study District with (**a**) linear y-axis, and (**b**) logarithmic y-axis.

Then, the distribution of urban area (the number of grids) against the number of fire events per grid under the 500-m grid size resolution system is summarized in Fig. 6. Totally, 4557 grids are generated for the Xiaoshan District at the 500-m resolution. A total of 4967 fire events are reported in the studied period, so a mean value of 1.09 fire per grid could be obtained. As shown in the figure, the frequency decreases as the number of fires per grid increases, showing a typical long-tail distribution. Meanwhile, 2592 grids have no fire reported and the maximum number of fires per grid is 25 (1 grid). This visualization supports the finding of spatial clustering, as it demonstrates a high occurrence of grids with few fires (namely, less than 5) and a progressively lower frequency of grids with many fires (larger than 10). The presence of a few grids with a significantly higher number of fires further suggests areas of intense fire activity within the district.

### Analysis model of fire influencing factors

Independence is a fundamental assumption of linear regression, requiring no perfect linear relationship between independent variables, a phenomenon known as collinearity. Collinearity is critical to address because its presence undermines the regression model’s interpretability and validity. Specifically, it makes it challenging to isolate the independent contribution of each variable to the dependent variable and leads to inflated standard errors of the regression coefficients, reducing the precision of parameter estimates. The Variance Inflation Factor (VIF), which quantifies how much the variance of a regression coefficient increases when other predictors are included in the model estimation method is adopted to validate the collinearity of the selected variables in the present study. Typically, a VIF value exceeding 10 is considered indicative of strong collinearity.

The collinearity is inherent in the data distribution due to the nature of the independent variables. Specifically, the proportions of all land-use types within a research grid sum to 1, creating a mathematical dependency among variables if all are included in the model. To address this, collinearity diagnostics are calculated and summarized in Table 4. The results reveal that the VIF for the variable “proportion of undeveloped land” is excessively high at 12,935.8, whereas the VIF values for other variables remain below 10. As a result, the “proportion of undeveloped land” variable was excluded from the regression model. This decision is further justified by its limited relevance to urban fire incidents, which are predominantly influenced by human activities. Undeveloped land, lacking significant human habitation or activity, is unlikely to meaningfully explain the occurrence of urban fires.

**Table 4:** Multicollinearity test results among selected indicators.

Variable	VIF
The proportion of residential land	1.142
The proportion of commercial land	1.171
The proportion of transportation land	1.048
The proportion of industrial land	1.065
The proportion of public administration and service land	1.098
The proportion of undeveloped land	12,935
Population density	1.450
GDP	7.64

After removing the collinear variable, the remaining proportions of land use types and population density could be regarded as independent variables, while the number of fires is designated as the dependent variable for negative binomial regression analysis. Throughout the regression process, variables with p-values less than 0.05 are identified, and those variables are eliminated before re-executing the regression analysis. The conclusive outcomes of the NBR regression analysis are summarized in Table 5.

As shown in the table, the regression coefficients for all variables are positive, indicating that higher levels of urban land development, regardless of type, are associated with an increased probability of fire occurrences. Notably, residential and commercial land use have the highest coefficients (4.482 and 3.384, respectively), highlighting their significant impact on fire frequency. This can be attributed, in part, to the intense human activity in these areas, including the frequent use of electrical appliances, cooking, and other activities that heighten fire risks. Additionally, residential and commercial buildings are generally more susceptible to fires compared to industrial and public service buildings. This vulnerability arises from differences in design and construction standards, such as the use of more flammable decorative materials and higher-density layouts. However, it is important to note that this study focuses solely on fire frequency. Industrial fires, although less frequent, often result in higher economic losses and greater casualties. Thus, a more comprehensive risk assessment is necessary, incorporating both the number of fire incidents and their potential consequences.

**Table 5:** Negative binomial regression results of selected indicators on fire incidents.

Variables	Coefficient	Standard error	z-Statistic	*P*-value
Constant	−2.265	0.087	−26.118	0.000
Proportion of residential land	4.482	0.141	31,849	0.000
Proportion of commercial land	3.384	0.329	7.429	0.000
Proportion of transportation land	1.914	0.226	8.492	0.000
Proportion of industrial land	2.906	0.137	21.213	0.000
Proportion of public administration and service land	1.644	0.265	9.965	0.000
Population density	3.686	0.835	9.928	0.000
GDP	0.470	0.147	3.197	0.001

Besides the land development, population density also presents a very significant positive correlation with the occurrence of urban fires (the coefficient of 3.686) with a relatively large standard error (0.835). This is phenomenological since human behavior is extremely complex. While population density as a quantitative indicator can reflect the degree of population aggregation, it cannot fully capture all the complex factors that may affect the occurrence of fires. There are many other factors, such as demographic structure, cultural level, safety awareness, economic and living conditions, that can also impact the likelihood of fire incidents. GDP is also positive and statistically significant, yet its promoting effect (the coefficient of 0.470) is the smallest among all included covariates. This likely reflects that much of the economic signal affecting urban fire frequency is already implicitly captured by population density and land-use composition (e.g., commercial/industrial shares).

### Model performance evaluation and comparison

To determine the most appropriate regression specification for our fire count data, we evaluated four candidate models: Zero-Inflated Negative Binomial (ZINB), Negative Binomial Regression (NBR), Poisson Regression, and Multiple Linear Regression (MLR). Their performance metrics are summarized in Table 6.

**Table 6:** Performance comparison of candidate regression models.

Model	pseudo-*R*²	AIC	Log-Likelihood	RMSE	MAE
ZINB	0.226	**9038.38**	−4717.19	2.33	1.13
NBR	**0.496**	9077.37	**−4530.68**	**1.99**	**1.06**
Poisson	0.352	11008.74	−5496.37	2.00	1.11
MLR	0.328	17097.55	−8540.77	2.25	1.12

The evaluation reveals a clear distinction between models designed for count data and the linear model. Both the ZINB and NBR models demonstrate significantly superior fit for our discrete, over-dispersed fire counts, as evidenced by their substantially lower AIC values (9038.38 and 9077.37, respectively) and higher log-likelihoods (−4717.19 and − 4530.68, respectively) compared to the Poisson and MLR models. This confirms that models specifically accounting for the discrete and over-dispersed nature of count data are essential for our analysis.

Between the two count-appropriate models, the Negative Binomial Regression (NBR) is selected as our final specification. Although the ZINB model has a marginally lower AIC, its pseudo-R² (0.226) is less than half that of the NBR model (0.496), indicating a markedly poorer explanation of the observed variance. This suggests that the zero-inflation component of the ZINB is likely superfluous for our data, where zero counts are adequately modeled by the NBR’s over-dispersion parameter. Furthermore, the NBR model achieves the best predictive accuracy, with the lowest RMSE (1.99) and MAE (1.06). Therefore, the NBR model provides the optimal combination of superior explanatory power, predictive accuracy, and theoretical appropriateness for over-dispersed count data.

### Fire risk map generation

**Fig. 7:**
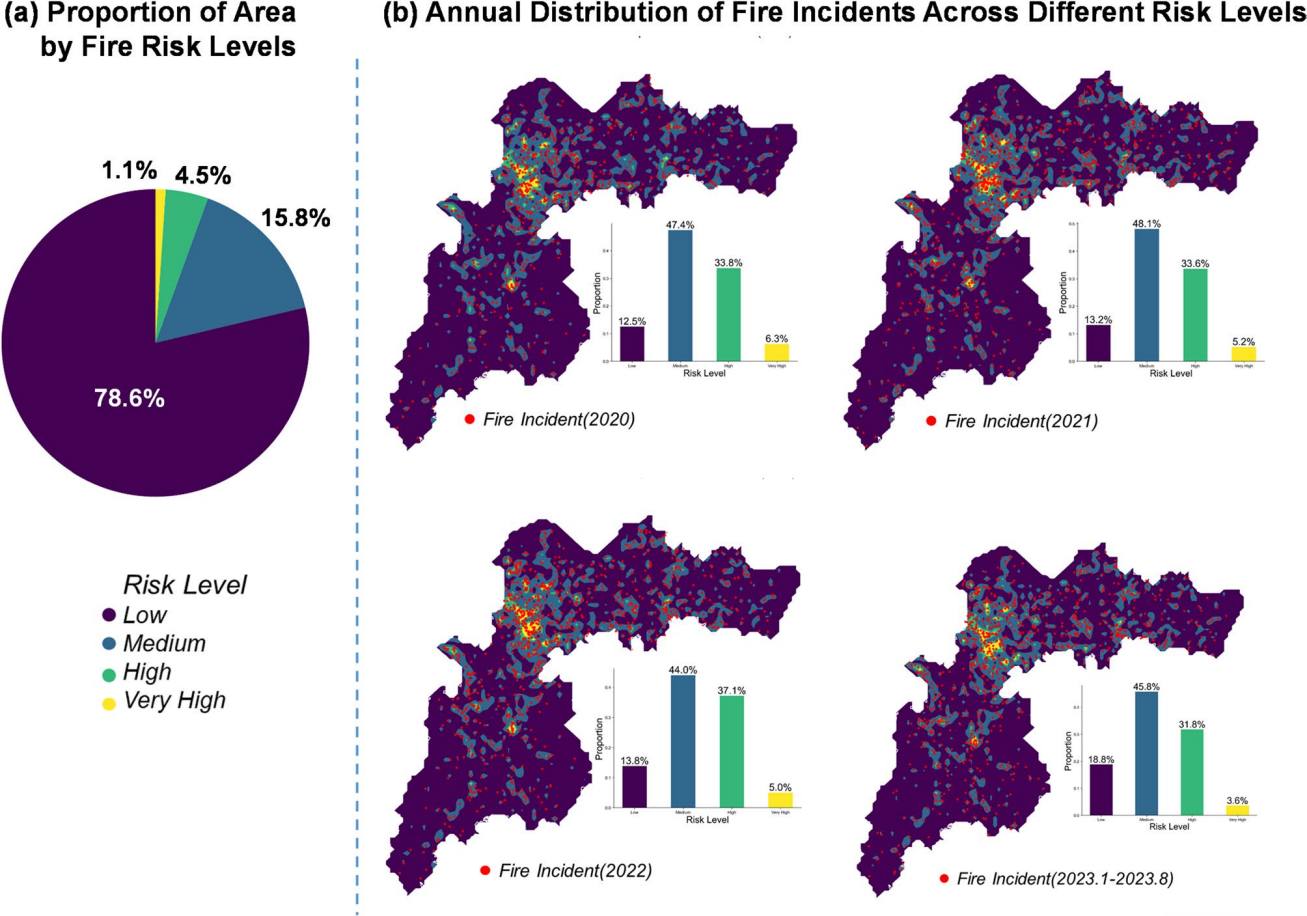
(**a**) Proportion of area by fire risk levels, and (**b**) Annual distribution of fire Incidents across different Risk Levels.

Using the proposed framework, the fire risk score could be calculated in each mesh of the target area, as shown in Fig. 7. Specifically, the negative binomial regression provides factor weights that reflect each variable’s marginal contribution to fire frequency; these weights are applied to the planning-oriented land-use shares and the traditional socioeconomic variables (population density and GDP). To classify fire risk levels, the natural breaks method^32^ is employed, dividing the computed risk values into four distinct categories: low, medium, high, and very high risk. The results reveal a compelling insight: while approximately 20% of the land area is classified as medium, high, or very high fire risk categories, this subset encompasses roughly 80% of the recorded fire incidents for all the 4-year period investigated. This observation aligns with the widely recognized “Pareto Principle” (80/20 rule)^33^ as described by Pareto, highlighting that a small proportion of high-risk urban areas accounts for the vast majority of fire incidents. Such a distribution underscores the spatial concentration of urban fire risks and the disproportionate vulnerability of specific zones.

This weight-based, hierarchical risk map thus ranks locations by structural vulnerability and provides a practical basis for prioritized, planning-oriented resource allocation.This discovery not only reinforces the spatial correlation patterns identified in earlier sections but also reveals a notable efficiency in targeting high-risk areas for urban fire management strategies. The alignment with the Pareto Principle provides an intriguing perspective on urban fire risk distribution, identifying the hotspot area in urban area. This finding highlights the potential to estimate fire risks in advance based on planned land use during the early stages of urban development. This methodology enables a detailed quantification of how different urban elements collectively influence fire incident likelihood. By leveraging this knowledge, urban planners can proactively identify high-risk zones and strategically allocate firefighting resources, such as equipment, infrastructure, and emergency response teams, to these areas. This targeted approach not only optimizes fire prevention strategies but also enhances urban resilience by integrating fire safety into the broader urban planning framework.

## Discussion

The analytical framework and empirical results presented in this study provide a data-driven foundation for understanding and mitigating urban fire risk. Moving beyond descriptive hotspot mapping, our approach quantifies the influence of planning-relevant factors—particularly fine-grained land-use composition—on fire frequency, and establishes a stable spatial structure for risk stratification. This section discusses the key implications of these findings for urban safety governance, spatial planning, and future research.

The 80/20 rule and the fire risk map provide a rigorous, data-driven basis for prioritizing fire prevention measures. By focusing efforts on these identified high-risk areas, resources for fire prevention, inspection, and community preparedness can be allocated with maximum efficiency. This ensures that investments—whether in infrastructure such as fire hydrants, regulatory enforcement, or public education—are deployed where they are most likely to reduce the total number and mitigate the severity of incidents. Furthermore, the static fire risk map generated in this study can serve as a foundational layer for an urban digital twin or smart firefighting system in the future. By integrating real-time dynamic data, it enables continuous, all-weather fire risk assessment.

An important empirical finding of this study is that residential land use and population density are the most critical factors influencing urban fire risk. This directly indicates that the focus of urban fire management should be on high-density residential areas, especially older, high-density neighborhoods with outdated facilities and inadequate fire safety standards. These areas require targeted facility upgrades, renovations, and enhanced fire safety supervision. At the same time, during urban renewal or new development, the quantitative risk coefficients provided by our model (such as the risk contribution of different land-use types) can be used to proactively assess the potential impact of planning schemes on regional fire risk. This contributes to shifting urban fire management from a predominantly “post-incident response” model to a proactive, risk-informed approach to resource planning.

Despite the valuable insights provided, this study has several limitations that should be acknowledged. The static analysis does not capture temporal risk variations such as seasonal or diurnal patterns, and the model’s parameters are calibrated specifically to the study area, which may limit direct generalizability to other urban contexts. Future research should develop spatiotemporal models by integrating time-series and real-time data, enabling dynamic risk forecasting and operational prevention. Furthermore, validating and adapting this framework across diverse cities will enhance its broader utility and support the evolution toward smarter, more resilient urban fire management systems.

## Conclusions

This paper presents an analytical framework for examining the spatial distribution characteristics and influencing factors of urban fires. The framework consists of three main components: the identification of influencing factors, with the novel inclusion of high-resolution land-use data alongside demographic variables; a spatial clustering analysis using Moran’s Index and the Silhouette Score to determine optimal grid size; and a regression analysis, leveraging negative binomial regression to quantify the impact of these factors on fire frequency.

Using Xiaoshan District as the empirical case, the negative binomial model demonstrates that, at the 500-m scale, population density, land-use composition, and GDP together explain roughly 50% of the spatial variation in fire occurrence. Among these variables, residential land is the strongest predictor; population density ranks second, followed by commercial land and other land-use types. These results highlight that both functional zoning and human aggregation fundamentally drive urban fire patterns. Based on the factor-weighted composite risk map, a clear Pareto-type structure emerges: approximately 20% of grids account for nearly 80% of recorded fires. This concentration pattern suggests that substantial gains in fire prevention can be achieved by focusing on a limited number of structurally vulnerable areas.

The findings provide a clear, data-driven basis for prioritizing fire prevention resources in the identified high-risk zones, integrating quantitative risk assessment into forward-looking urban planning, and using the static risk map as a foundation for dynamic management systems.

## Supplementary Information

Below is the link to the electronic supplementary material.

Supplementary Material 1

## Data Availability

The datasets used and/or analysed during the current study are available from the corresponding author on reasonable request.
