# Supplementary material for: Risk maps for urban fire with geospatial model-based framework
Source: Sci Rep. 2026 Feb 7;16:7702. doi: 10.1038/s41598-026-38373-2 (PMC12946170; doi:10.1038/s41598-026-38373-2)
Supplement: Supplementary file 1 — Supplementary Material 1 [file 41598_2026_38373_MOESM1_ESM.docx]

**Supplementary Material: Calculation of the Silhouette Score**

The Silhouette Score is a metric used to evaluate the quality of clustering by measuring how similar an object is to its own cluster compared to other clusters. It provides a way to assess the appropriateness of the chosen spatial grid size by evaluating the cohesion and separation of the spatial clusters of grid cells identified through LMI analysis. The Silhouette Score *s*(*i*) for a single sample is defined as bellow:

| $s\left( i \right)=\frac{b\left( i \right)-a(i)}{max\left\{ a\left( i \right),b\left( i \right) \right\}}$ | $(1)$ |
| --- | --- |

where *a*(*i*) is the average distance between the *i*-th grid cell and all other grid cells in the same LMI cluster, and *b*(*i*) is the average distance between the *i*-th grid cell and all grid cells in the nearest neighboring LMI cluster. The Silhouette Score ranges from -1 to 1. A score close to 1 indicates that a grid cell’s fire count is very similar to others in its assigned LMI cluster and distinct from those in the nearest other LMI cluster. The average Silhouette Score across all grids thus quantifies how well-separated and internally consistent the LMI-derived spatial patterns (hot spots, cold spots, etc.) are at a given grid scale. This metric was used comparatively across grid sizes to identify the scale that produced the most coherent spatial clustering structure.
